# Nonlinear Dynamics and Entropy of Complex Systems with Hidden and Self-Excited Attractors

**DOI:** 10.3390/e21040370

**Published:** 2019-04-05

**Authors:** Christos K. Volos, Sajad Jafari, Jacques Kengne, Jesus M. Munoz-Pacheco, Karthikeyan Rajagopal

**Affiliations:** 1 Laboratory of Nonlinear Systems, Circuits & Complexity (LaNSCom), Department of Physics, Aristotle University of Thessaloniki, Thessaloniki 54124, Greece; 2 Nonlinear Systems and Applications, Faculty of Electrical and Electronics Engineering, Ton Duc Thang University, Ho Chi Minh City 700000, Vietnam; 3 Department of Electrical Engineering, University of Dschang, P.O. Box 134 Dschang, Cameroon; 4 Faculty of Electronics Sciences, Autonomous University of Puebla, Puebla 72000, Mexico; 5 Center for Nonlinear Dynamics, Institute of Research and Development, Defence University, P.O. Box 1041 Bishoftu, Ethiopia

**Keywords:** hidden attractor, complex systems, fractional-order, entropy, chaotic maps, chaos

In the last few years, entropy has been a fundamental and essential concept in information theory. It is also often used as a measure of the degree of chaos in systems; e.g., Lyapunov exponents, fractal dimension, and entropy are usually used to describe the complexity of chaotic systems. Thus, it will be important to study entropy in nonlinear systems. Additionally, there has been an increasing interest in a new classification of nonlinear dynamical systems including two kinds of attractors: self-excited attractors and hidden attractors. Self-excited attractors can be localized straightforwardly by applying a standard computational procedure. Some interesting examples of systems with self-excited attractors are chaotic systems with different kinds of symmetry, with multi-scroll attractors, with multiple attractors, and with extreme multistability.

In systems with hidden attractors, we have to develop a specific computational procedure to identify the hidden attractors because the equilibrium points do not help in their localization. Some examples of this kind of system are chaotic dynamical systems with no equilibrium points, with only stable equilibria, with curves of equilibria, with surfaces of equilibria, and with non-hyperbolic equilibria. There is evidence that hidden attractors play a vital role in various fields ranging from phase-locked loops, oscillators, describing convective fluid motion, a model of the drilling system, information theory and cryptography to multilevel DC/DC converters. Furthermore, hidden attractors may lead to unexpected and disastrous responses.

The overall purpose of this Special Issue lies in gathering the latest scientific trends on the advanced topics of dynamics, entropy, fractional-order calculus, and applications in complex systems with hidden attractors and self-excited attractors.

In the paper “A New Chaotic System with Multiple Attractors: Dynamic Analysis, Circuit Realization and S-Box Design”, Qiang Lai, Akif Akgul, Chunbiao Li, Guanghui Xu, and Ünal Çavuşoğlu report a novel three-dimensional chaotic system with three nonlinearities. The system has one stable equilibrium, two stable equilibria, and one saddle-node, two saddle foci and one saddle-node for different parameters. Also, an electronic circuit is given for implementing the chaotic attractors of the system, and an S-Box is developed for cryptographic operations [1].

In the paper “A New Chaotic System with a Self-Excited Attractor: Entropy Measurement, Signal Encryption, and Parameter Estimation”, Guanghui Xu, Yasser Shekofteh, Akif Akgül, Chunbiao Li and Shirin Panahi introduce a new chaotic system with an engineering application for signal encryption. The implementation and manufacturing are performed via a real circuit as a random number generator. Also, the authors provide a parameter estimation method to extract chaotic model parameters from the real data of the chaotic circuit using a Gaussian mixture model (GMM) and two optimization algorithms: WOA (Whale Optimization Algorithm), and MVO (Multi-Verse Optimizer) [2].

In the paper “A Novel Algorithm to Improve Digital Chaotic Sequence Complexity through CCEMD and PE”, Chunlei Fan, Zhigang Xie, and Qun Ding introduce a three-dimensional chaotic system with a hidden attractor. The complex dynamic behaviors of the system are analyzed by Poincaré cross-sections, equilibria, and initial values. Further, they have designed a new algorithm based on complementary ensemble empirical mode decomposition (CEEMD) and permutation entropy (PE) that can effectively enhance digital chaotic sequence complexity [3].

In the paper “A New Two-Dimensional Map with Hidden Attractors”, Chuanfu Wang and Qun Ding investigate the hidden dynamics of a new two-dimensional map inspired by Arnold’s cat map and study the existence of fixed points and their stabilities in detail [4].

In the paper “Stochastic Entropy Solutions for Stochastic Nonlinear Transport Equations”, Rongrong Tian and Yanbin Tang analyze the existence and uniqueness of the stochastic entropy solution for a nonlinear transport equation with a stochastic perturbation. They prove the continuous dependence of stochastic robust entropy solutions on the coefficients and the nonlinear functions [5].

In the paper “Multivariate Multiscale Complexity Analysis of Self-Reproducing Chaotic Systems”, Shaobo He, Chunbiao Li, Kehui Sun and Sajad Jafari propose a chaotic system with infinitely many attractors. Multiscale multivariate permutation entropy (MMPE) and multiscale multivariate Lempel–Ziv complexity (MMLZC) are employed to analyze the complexity of these self-reproducing chaotic systems with infinitely many chaotic attractors [6]. 

In the paper “A New Fractional-Order Chaotic System with Different Families of Hidden and Self-Excited Attractors”, Jesus M. Munoz-Pacheco, Ernesto Zambrano-Serrano, Christos Volos, Sajad Jafari, Jacques Kengne and Karthikeyan Rajagopal introduce a new fractional-order chaotic system with a single parameter and four nonlinearities. One striking feature is that by varying the system parameter, the fractional-order system generates several complex dynamics: self-excited attractors, hidden attractors, the coexistence of hidden attractors, and multistability. Moreover, the complexity of the system is analyzed by computing its spectral entropy and Brownian-like motions [7].

In the paper “A New Chaotic System with Stable Equilibrium: Entropy Analysis, Parameter Estimation, and Circuit Design”, Tomasz Kapitaniak, S. Alireza Mohammadi, Saad Mekhilef, Fawaz E. Alsaadi, Tasawar Hayat and Viet-Thanh Pham present a new three-dimensional chaotic system with one stable equilibrium. This system is a multistable dynamic system in which the strange attractor is hidden. To show the feasibility and ability in engineering applications of the proposed system, an entropy analysis, parameter estimation, and circuit design are performed [8].

In the paper “Optimization of Thurston’s Core Entropy Algorithm for Polynomials with a Critical Point of Maximal Order”, Gamaliel Blé and Domingo González discuss some properties of the topological entropy systems generated by polynomials of degree *d* in their Hubbard tree. An optimization of Thurston’s core entropy algorithm is developed for a family of polynomials of degree *d* [9].

In the paper “Strange Attractors Generated by Multiple-Valued Static Memory Cell with Polynomial Approximation of Resonant Tunneling Diodes”, Jiri Petrzela studies the multiple-valued memory system (MVMS) composed by a pair of resonant tunneling diodes (RTD). For specific values of system parameters, such a tunnel shows a double-spiral chaotic attractor. The existence of these types of strange attractors is proved using the largest Lyapunov exponents (LLE) and computer-aided simulation of the designed lumped circuit using only commercially available active elements [10].

In the paper “The Co-existence of Different Synchronization Types in Fractional-order Discrete-time Chaotic Systems with Non–identical Dimensions and Orders”, Samir Bendoukha, Adel Ouannas, Xiong Wang, Amina-Aicha Khennaoui, Viet-Thanh Pham, Giuseppe Grassi, and Van Van Huynh analyze the co-existence of different synchronization types for fractional-order discrete-time chaotic systems with different dimensions. They show that through appropriate nonlinear control, projective synchronization (PS), full state hybrid projective synchronization and inverse full state hybrid projective synchronization (IFSHPS), generalized synchronization is achieved [11].

In the paper “The Complexity and Entropy Analysis for Service Game Model Based on Different Expectations and Optimal Pricing”, Yimin Huang, Xingli Chen, Qiuxiang Li and Xiaogang Ma propose a multichannel dynamic service game model to analyze the relations between the manufacturer and the retailer under optimal pricing. Theoretical analysis of the model and numerical simulations from the perspective of entropy theory, game theory, and chaotic dynamics are conducted. Chaotic and complex behaviors are observed causing the system’s entropy to increase when the manufacturer adjusts the service decision quickly [12].

In the paper “Dynamics and Complexity of a New 4D Chaotic Laser System”, Hayder Natiq, Mohamad Rushdan Md Said, Nadia M. G. Al-Saidi and Adem Kilicman introduce a new 4D chaotic laser system with three equilibria and only two quadratic nonlinearities. Dynamics analysis, including stability of symmetric equilibria and the existence of coexisting multiple Hopf bifurcations on these equilibria, are investigated. Moreover, the complexity of the laser system reveals that system time series can locate and determine the parameters and initial values that show coexisting attractors [13].

In the paper “Entropy Analysis and Neural Network-Based Adaptive Control of a Non-Equilibrium Four-Dimensional Chaotic System with Hidden Attractors”, Hadi Jahanshahi, Maryam Shahriari-Kahkeshi, Raúl Alcaraz, Xiong Wang, Vijay P. Singh, and Viet-Thanh Pham present a non-equilibrium four-dimensional chaotic system with hidden attractors. Its dynamical behavior is investigated using a bifurcation diagram, as well as three well-known entropy measures: approximate entropy, sample entropy, and Fuzzy entropy. Additionally, an adaptive radial-basis-function neural network (RBF-NN)-based control method is proposed [14].

In the paper “Adaptive Synchronization of Fractional-Order Complex Chaotic system with Unknown Complex Parameters”, Ruoxun Zhang, Yongli Liu and Shiping Yang investigate the problem of synchronization of fractional-order complex-variable chaotic systems (FOCCS) with unknown complex parameters. Based on the complex-variable inequality and stability theory for fractional-order complex-valued systems, a new scheme is presented for adaptive synchronization of FOCCS with unknown complex parameters [15].

In the paper “Chaotic Map with No Fixed Points: Entropy, Implementation and Control”, Van Van Huynh, Adel Ouannas, Xiong Wang, Viet-Thanh Pham, Xuan Quynh Nguyen, and Fawaz E. Alsaadi propose a map without equilibrium. The map has no fixed point but exhibits chaos. The entropy of this new map has been calculated. Also, experimental observations of the map using an open micro-controller platform are given [16].

In the paper “Dynamics and Entropy Analysis for a New 4-D Hyperchaotic System with Coexisting Hidden Attractors”, Licai Liu, Chuanhong Du, Xiefu Zhang, Jian Li, and Shuaishuai Shi report a new no-equilibrium 4-D hyperchaotic multistable system with coexisting hidden attractors. One prominent feature is that by varying a system parameter or initial value, the system can generate several nonlinear complex attractors: periodic, quasiperiodic, multiple topologies chaotic, and hyperchaotic [17].

The Guest Editors hope that you will enjoy reading this Special Issue devoted to this exciting and fast-evolving field, and that it will motivate researchers to pursue further advances in the emerging areas of complex systems with hidden and self-excited attractors.
